# The role of the University of Padua medical school in the study of conjoined twins between 18th and early 19th century

**DOI:** 10.1002/ajmg.a.62938

**Published:** 2022-08-10

**Authors:** Giovanni Magno, Lucas L. Boer, Roelof‐Jan Oostra, Alberto Zanatta

**Affiliations:** ^1^ University Museums Centre CAM, University of Padua Padua Italy; ^2^ Department of Imaging, Section Anatomy and Museum for Anatomy and Pathology Radboud University Medical Center Nijmegen The Netherlands; ^3^ Department of Medical Biology, Section Clinical Anatomy and Embryology Amsterdam University Medical Centers, University of Amsterdam Amsterdam The Netherlands; ^4^ Department of Cardiac, Thoracic, Vascular Sciences and Public Health University of Padua Padua Italy

**Keywords:** conjoined triplets, conjoined twins, museum, Paduan medical school, teratology

## Abstract

The Medical School of Padua (Italy) contributed profoundly to the study of teratology. Many famous physicians and professors of medicine, such as Liceti, Vallisneri, Morgagni, and Malacarne, have studied and investigated these anomalies to better understand the causes and to find a potential explanation, often preserving the specimens for future studies. The present study highlights some historical cases of conjoined twins and a conjoined triplet preserved at the Morgagni Museum of Human Anatomy to show the development of medical theories in the teratological field between the 18th and early 19th century. This approach will provide insights into different study methods and ideas of some of the most famous scholars working in Padua at that time. The current article focuses on rare cases, both human and animal, that were encountered by physicians who worked in the Veneto area in the late 18th and early 19th century. Their detailed descriptions are not only of historical but also of contemporary dysmorphological value.

## 1 INTRODUCTION

The University of Padua holds a very long tradition that has made it one of the most famous universities in the world, because of the enormous contributions of scholars in medicine, anatomy, philosophy, and pathology, thanks to professors as Andreas Vesalius (1514–1564), father of Anatomy, Hieronymus Fabricius (1533–1619), and Giovanni Battista Morgagni (1682–1771), father of Pathological Anatomy and many more. It was in fact here that there was the first scientific approach to congenital malformations. This article provides an historical review of some of the most important scholars of Padua and their seminal works in this field, also providing illustrative “cases,” and focusing on conjoined twins, which should be of interest to human and animal geneticists interested in teratology, dysmorphology, pathology, perinatal medicine, and the depiction of birth defects in medical illustrations (Ongaro, 2008; Tekendo‐Ngongang et al., 2021; Zanatta et al., 2018).

## 2 HISTORICAL BACKGROUND

### 2.1 The 17th century

The University of Padua Medical School holds a very long tradition that has made it one of the most famous universities in the world, thanks to professors as Andreas Vesalius (1514–1564), father of Anatomy, Hieronymus Fabricius (1533–1619), and Giovanni Battista Morgagni (1682–1771), father of Pathological Anatomy. Among them, is also Fortunio Liceti (1577–1657), professor of Philosophy and Theoretical Medicine in Padua, and one the first to profess a scientific approach toward congenital malformations.

Liceti was a follower of Aristotelian principles, but differed from him in the theories on generation. He assumed the existence of a “female seed” in addition to the male one, both made up of residual blood coming from all parts of the body, of which they retain traces. This theory, derived from Hippocrates, Democritus, and Galen, was propagated by Liceti to explain embryonic development, in particular the hereditary transmission of characteristics, hybridization and the production of monsters, considered instead by the Aristotelians to be related to supernatural causes or crossbreeding between species (Ongaro, 2008).

In 1616, Liceti published the *De Monstrorum caussis natura et differentiis* in Padua, a turning point in the study of congenital malformations (Liceti 1616). In this treatise, a classification is conducted according to, then valid, morphological criteria leading to a wide variety of anomalies. Apparently, his criteria for inclusion were so profound that much of the congenital malformations known today were described (Ongaro, 2008). Liceti was the first to consider natural causes for the occurrence of malformations, such as compression of the membranes in the uterus, abnormalities of the placenta or amniotic fluid, malnutrition, trauma, or fetal disease. Nevertheless, in line with his contemporaries, he still considered “moral” issues, ranging from “*phantasia*” and “*animi passionibus*,” that is, imaginations of the parents at the time of conception or during pregnancy, up to the “*malos Daemones*,” evil influences, as legitimate explanations (Fulcheri, 2002).

### 2.2 The 18th century

After Liceti, several scholars of the Padua University continued his research on teratology, in particular Antonio Vallisneri (1661–1730), Giovanni Battista Morgagni, Vincenzo Malacarne (1744–1816), and Jacopo Penada (1748–1828).

Vallisneri, professor in Padua of Practical Medicine and subsequently Theoretical Medicine, distinguished himself for his studies in the field of human pathology, supported by comparative naturalistic and anatomical studies as a basis for medical studies (Galassi et al., 2018). He observed several cases of monstrosities and theorized that conjoined twins were born “*from two germs or mature eggs, which, by matching closely, over time attack and interpenetrate, so that they compose a doubled body. This seems evident in the hen*'*s eggs, which have two or more yolks, from which chickens with two or more heads, or with multiplied limbs, are born. Instead, the twins are born separated, when the eggs are separated in the trumpets, so they descend in the same way into the matrix and each has its own placenta, its umbilical vessels and its enveloping membranes*” (Vallisneri, 1721). Vallisneri and Liceti in fact were also among the few to discover and preserve two of the five known cases, as per late 19th century, of asymmetric or heteropagus conjoined twins (Taruffi, 1899).

Morgagni, successor of Vallisneri to the chair of Theoretical Medicine in 1711 and chair of anatomy in 1715, was the first to consider congenital malformations exclusively as natural phenomena. Both Morgagni and Vallisneri tried to find logical explanations for fetal conditions, including “extraordinary” anomalies and deformations of the organs. Morgagni complained of the difficulty in understanding the causes of these malformations, which was impeded by the inability to carry out adequate autopsies on the fetuses, not only because the parents refused, but also for the lack of skills among the dissectors, who were not trained for these types of autopsies. Morgagni considered the study of these anomalies fundamental for the advance of medical science, but nonetheless difficult to carry out properly without autopsies (Morgagni, 1761). Both Morgagni and Vallisneri observed various cases of conjoined twins, anencephaly, spina bifida, and hydrocephalus, in both humans and animals. Some of these cases were preserved as museal specimens (Taruffi, 1882).

### 2.3 The 19th century

Malacarne arrived in Padua as professor of Theoretical and Practical Surgery and of Clinical and Surgical Operations in 1794 and moved to the chair of Surgical Institutions and Obstetrics in 1806. Finally, he was appointed director of the Obstetric Museum of the University, the collection of which partially remains preserved in the Morgagni Museum of Human Anatomy and the Department of Women's and Children's Health. Starting from 1806, he studied congenital monstrosities and malformations with the aim to create a new classification, which was based on the external form of the body. Part of the nomenclature he introduced is still used today (Ongaro, 2017; Zanatta et al., 2018).

Contemporary to Malacarne was Jacopo Penada (1748–1828), physician of the Departmental Health Commission of Padua. Penada was a dissector at the University of Padua and honorary professor of the University of Vilnius (Penada, 1804). Additionally, he was employed as curator and custodian of the old Pathological Museum of the University of Padua. Aside from studying various human and animal teratological cases, he also took care of preparing and preserving the specimens present in the Museum (Malacarne, 1807).

It was only in the 1820s that Etienne Geoffroy Saint‐Hilaire (1772–1844), French naturalist and founder of comparative anatomy, coined the term “teratology” as “science of monsters” (Saint‐Hilaire, 1832). Saint‐Hilaire was one of the first to highlight the educational value of “monsters,” classifying them and trying to establish their origin, even with experimental attempts on chicken eggs (DeSesso, 2019; Morin, 1996).

Between the 18th and 19th century, the renewed interest in congenital anomalies and especially in conjoined twins led many scholars to study them scientifically, including Nicola Bongiovanni (n.d.) and his son Zenone (n.d.), both physicians from Verona, and Francesco Fanzago (1764–1836), professor of Pathology and Rector of the University. Bolognese pathologist Cesare Taruffi (1821–1902) in his monumental multivolume masterpiece about the history of teratology (“*Storia della Teratologia*”) also reported these specimens and their peculiarities. Taruffi's work led to the development of a school of teratological studies in Bologna (Scarani & Eusebi, 2009).

Lodovico Brunetti (1813–1899) gathered a substantial number of teratological specimens since the 1860s. Brunetti became the first chair of Pathological Anatomy in Padua in 1855 and felt the need to realize a Museum of pathology, as he was aware of the importance of pathological preparation in the training of medical students. To nourish this need, he started to preserve and expose different specimens. In addition to his collecting urge, he used innovative techniques to preserve his acquired specimens through peculiar human taxidermy (Magno et al., 2021) and via his own technique of tannization, an artificial mummification using tannic acid injections (Magno et al., 2021; Zanatta & Zampieri, 2018).

In 2018, the Museum founded by Brunetti has been renewed and renamed Morgagni Museum of Human Anatomy, Section of Anatomical Pathology. All its collections testify to the strong medical tradition of Padua University, especially regarding the field of teratology. Currently, ~100 teratological specimens are still present in the extant pathological collection of the Morgagni Museum (Zanatta & Zampieri, 2018).

The current research will analyze some of the oldest specimens of conjoined twins, both human and animal, present in the Morgagni Museum collection, in order to shed new light on these historical cases. They are dated between the late 18th and early 19th century. The case descriptions presented here are based on translations of the original Latin and Italian texts and exemplify the evolution of medical knowledge on these congenital anomalies. Although in scientific literature the term “monsters” has attained a derogatory connotation and has been replaced by “birth defects” or “congenital anomalies” since 1958 (Dron, 2016), we decided to stay close to the original terminology, thus reflecting the evolution of medical‐cultural knowledge and language.

## 3 CASES REPORTS

We will use the terms “autosite” and “parasite” to refer to the independent and dependent co‐twins, respectively.

### 3.1 Case 1. Antonio Vallisneri (1718) and the parasitic conjoined twin

Vallisneri (1721) reported on one of the first cases known from Padua of a peculiar parasitic conjoined twin in his book *Istoria della generazione*. He also stated as Morgagni commented the case as an interesting aberration, useful to understand natural variability, but he did not deal with the case, leaving it to Vallisneri (1721).

**FIGURE 1:**
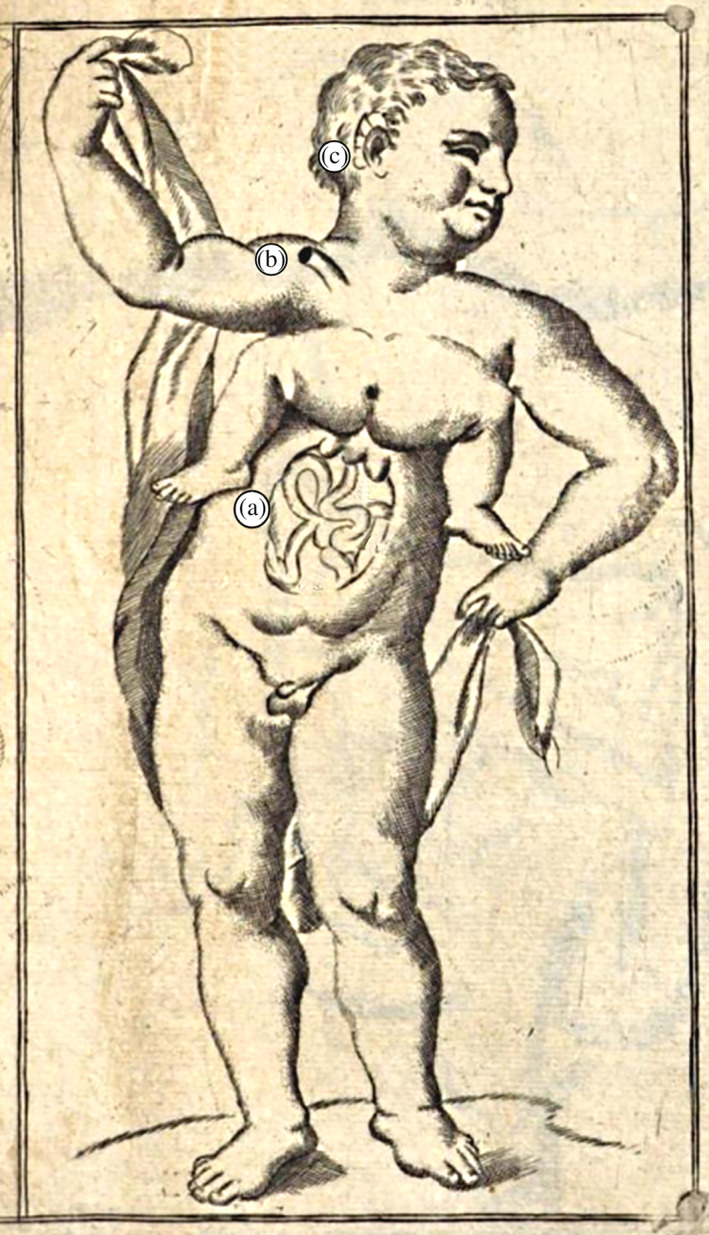
Male child with parasitic twin. (a) Large herniation at the level of the umbilicus; (b) an exiting vessel, like an umbilical cord; (c) another small ear on the normal one. Drawing from Vallisneri (1721) and edited by the authors

The Vallisneri case concerned male parasitic conjoined twins delivered in the seventh month of pregnancy in Bologna on November 9, 1718. The child presented a herniation of the abdominal organs, scarcely retained by the peritoneum (Figure 1a). The child died after 2 days and Antonio Sebastiano Trombelli (n.d.), a Bolognese physician, performed an autopsy albeit limited 4 h after the death. Unfortunately, only one drawing of this case made by Vallisneri survived, whereas the original specimen has been lost.

The description of the case reported here in a translation from the Latin original to clarify the peculiarity of this condition. Vallisneri started his description by mentioning that, in addition to a large umbilical hernia, there was *“an almost dried curved vessel coming out like an umbilical cord”* running out from under the right clavicle (Figure 1b). The right eye fell out naturally, allowing one to see clearly inside the inner parts of the empty orbit. Near the right eye, there were two supernumerary ears, above which two small holes were present on each side, giving entrance to a winding duct that ran in the direction of the tympanic cavity (Figure 1c). Attached to the upper part of the autosite's sternum was the third lumbar vertebra of the parasite, who further consisted only of buttocks with a sacrum, femurs, and tibias. The parasite's anus was closed, the scrotum lacked testicles and was divided into two cavities. A toe on its left foot was missing.

After opening the abdomen, the intestines showed an unusual rotation, with a potential necrotic herniation described as a small, dark stained portion of the umbilical hernia protruding through the omentum. The stomach was filled with milk and cooked apple. The intestines between the stomach and the middle part of the mesentery were normal.

Vallisneri mentioned that the intestines included duplications, probably of the ileum and colon, as well blind ending parts and connections with the urinary system.

There was a normal liver located on the right side of the abdomen while the one on the left was smaller and showed a more reddish color. The spleen was larger than normal but in general, it had a completely natural condition, just like the pancreas. The left kidney seemed to be formed by five different small globular kidneys and it was about a third of its size larger than normal. The right kidney had a similar globular aspect.

The parasite showed an exposed pelvis facing the body of the autosite, and communicating with its chest. There was one dysfunctional bladder, one kidney and two testicles, the right one being larger than the left. The supernumerary end of the small intestine previously described communicated with the bladder in an unclear manner, as did the vessel‐like structure, which came out from below the right clavicle.

There were two hearts, one on each side of the chest, with a single pericardium at the top, splitting subsequently in two parts. The left heart had a normal size. Toward the apex the two ends of the hearts both extended laterally. The brain, instead, did not present anything abnormal. This condition was diagnosed as asymmetric prosopothoracoileopagus conjoined twins (Oostra et al., 2022), complicated by an omphalocele and what seemed to have been rectovesical fistulas.

### 3.2 Case 2. Nicola Bongiovanni and the conjoined twins (1768)

**FIGURE 2:**
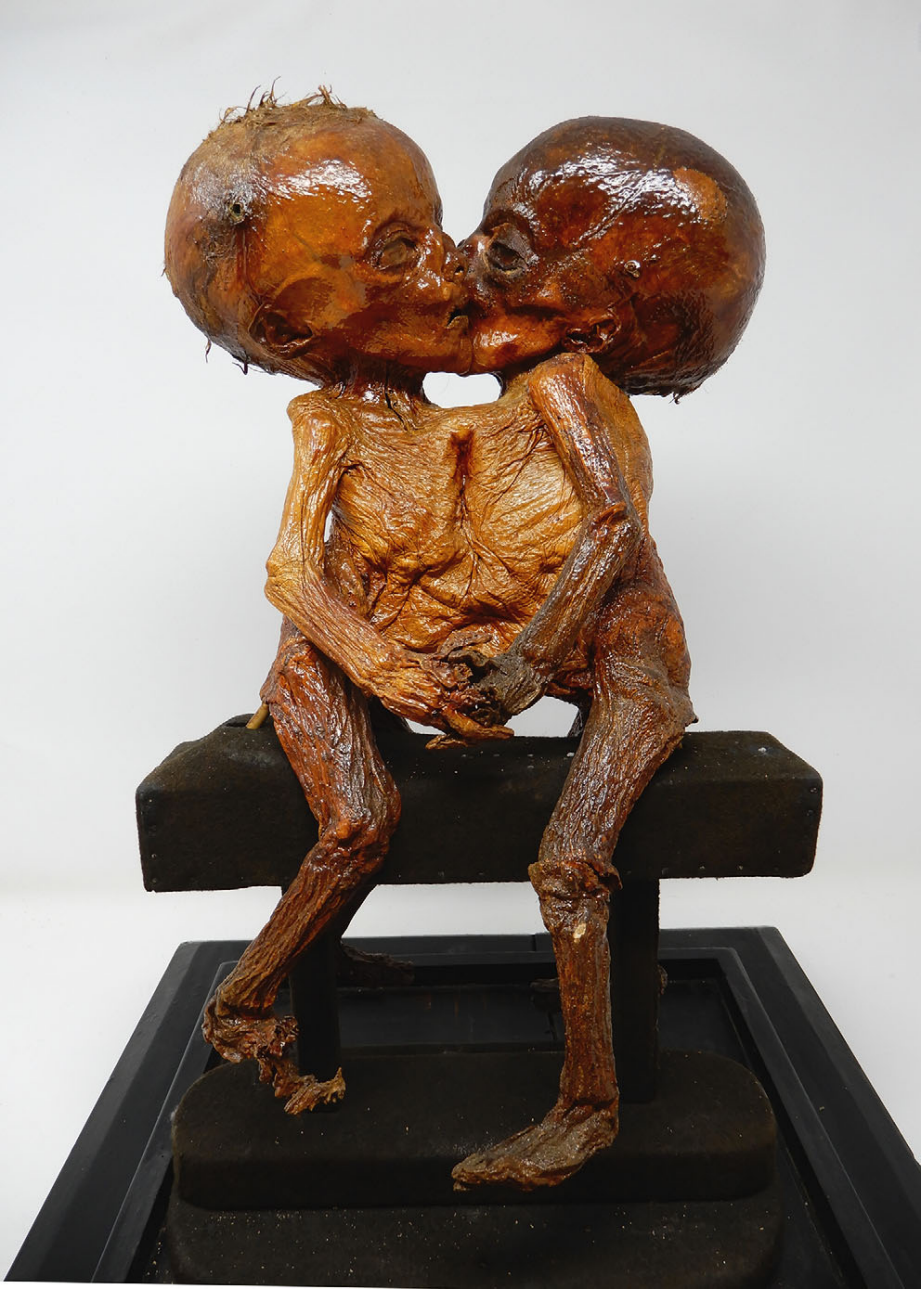
Embalmed female conjoined twins specimen corresponding to Nicola Bongiovanni's description of 1769 from Morgagni Museum

In his treatise of 1769 about variolation, Nicola Bongiovanni mentioned a specimen in his possession of two conjoined female twins “*of extraordinary size and perfect structure, joined together wonderfully from the clavicles up to the umbilicus*” (Figure 2). The twins were born on July 15, 1768 and died soon after birth. Their bodies were sent to Bongiovanni for autopsy and preservation for further studies (Bongiovanni, 1769).

In the pathological collection of the Morgagni Museum, there is a specimen corresponding to Bongiovanni brief description: female ventrally conjoined twins, joined at their thoraxes, and abdomens. The mummified specimen does not show further macroscopic alterations. We diagnosed the condition as symmetric thoracoileopagus conjoined twins. Since the description by Bongiovanni did not include any pictures, and the specimen lacks specific physical characteristics, a match between the two could not be made with complete certainty. The state of conservation of the specimen and the type of mummification, different from Brunetti's tannization (Zanatta & Zampieri, 2018), tend to confirm a potential correlation between the specimen itself and the cited case. Nevertheless, a further specimen was studied by Bongiovanni's son and it is still present in the collection, as it will be described later, thus assuming a potential family donation to the ancient pathological museum of the University of Padua.

### 3.3 Case 3. Zenone Bongiovanni and the “*monstrous child*” (1789)

**FIGURE 3:**
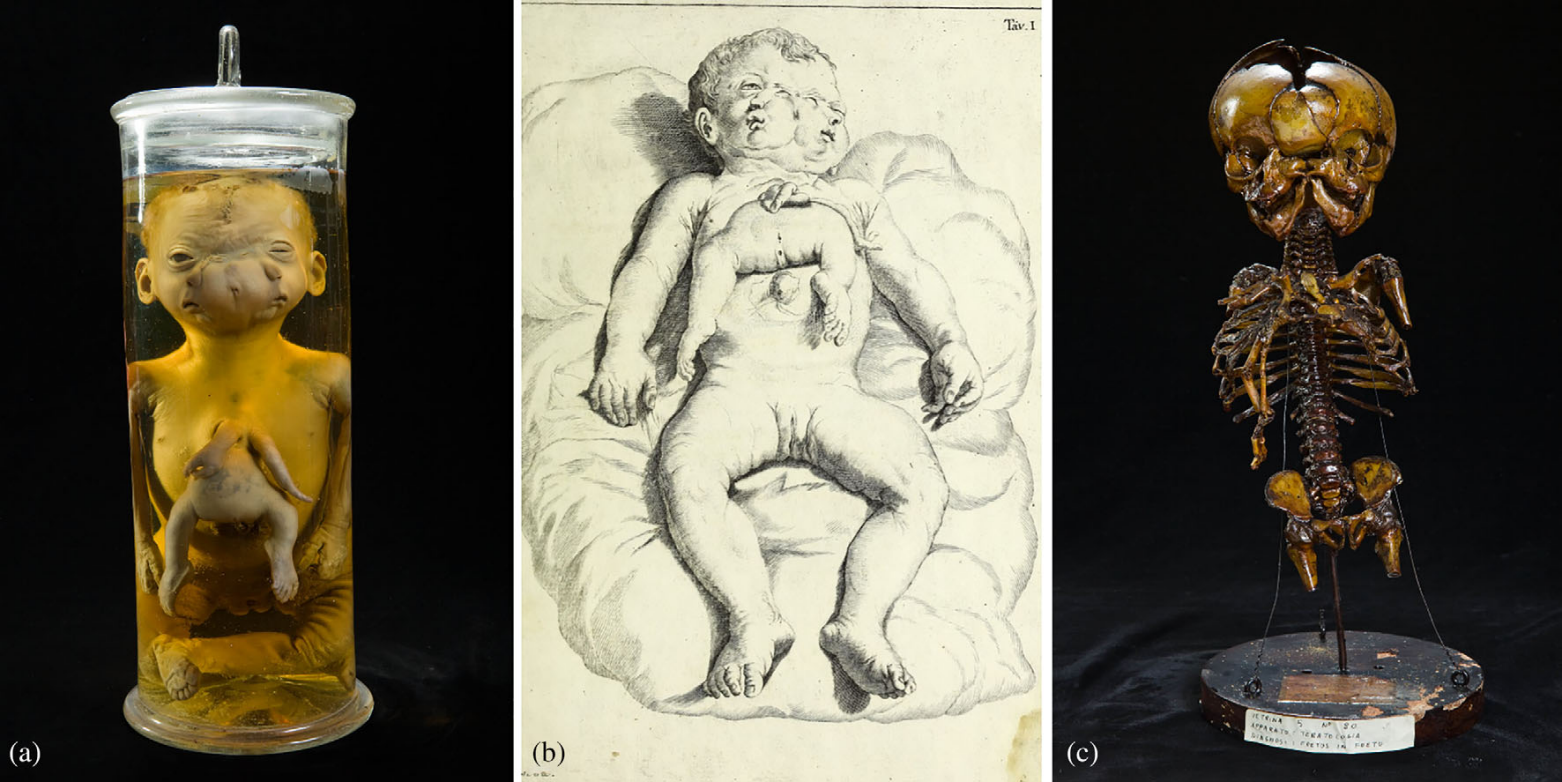
(a) Female conjoined twins with parasitic third twin on the chest from Morgagni Museum. (b) Drawing ordered by Zenone Bongiovanni portraying the individual at the time of death (1789). (c) Skeleton of the female conjoined twins

In 1789, Zenone Bongiovanni, son of Nicola, described a case of a female fetus (Figure 3), born spontaneously after an uneventful pregnancy, with two symmetrical faces (species *diprosopus triophthalmus* according to Taruffi, 1882) who had a headless parasite with four limbs, attached to its sternum (Bongiovanni, 1789).

As described by Bongiovanni, both mouths produced moans and whines, even at different pitch and the lateral eyes managed to open and move on their own. All parts of the body, including those of the parasite, were stated to show spontaneous movements. However, attempts of breastfeeding were unsuccessful and death occurred after only 2 days.

The specimen was “taxidermized” and preserved in liquid. The skeleton was dried for further study. Both are still preserved in the collection of Morgagni Museum, with a classification of “fetus in fetu”.

The autopsy, performed by a team of surgeons of the Public Health Office of Verona led by his father Nicola, reported that the individual was quite well‐formed, however an almost completely duplicated face and a ventrally inserted parasitic twin were clearly noticeable. Internal organs of the autosite, including the heart and liver, did not present with any abnormality, except for the intestines, connected to those of the parasite and partially protruding in the autosite's thorax resulting from a diaphragmatic hernia.

The parasite member consisted only of a headless trunk with rudimentary intestines, arms and legs. Furthermore, the central part of the partially duplicated faces of the autosite created the illusion of a third face, made in fact by conjoined small inward eye opening and conjunction of the zygomatic arches and external auditory meatuses, as can be concluded from examining the skull.

The condition of this specimen is to be interpreted as laterally conjoined twins (parapagus diprosopus), forming a ventral conjunction with a third parasitic individual, which makes this a truly unique case of asymmetric conjoined triplets (Oostra et al., 2022).

### 3.4 Case 4. Fanzago and the “*bicorporeal monster*” (1803)

Fanzago reported on a case of female conjoined twins born naturally but prematurely in the seventh month of pregnancy on November 6, 1802. There was only one placenta and one umbilical cord; the placenta had a bigger size than normal. They presented with an extensive osseous and membranous union in most of the abdomen and part of the thoraxes. Despite their conjunction, the twins were born in breech position after 3 hours of labor. A slight deformation of the fronto‐parietal bones was reported due to prolonged compression during labor. At the time, it was common for deliveries of conjoined twins to occur naturally, also considering that cesarean section techniques that were not fatal for the mother were not practiced before the end of the 19th century (Fanzago, 1803; Mazzarello, 2017).

**FIGURE 4:**
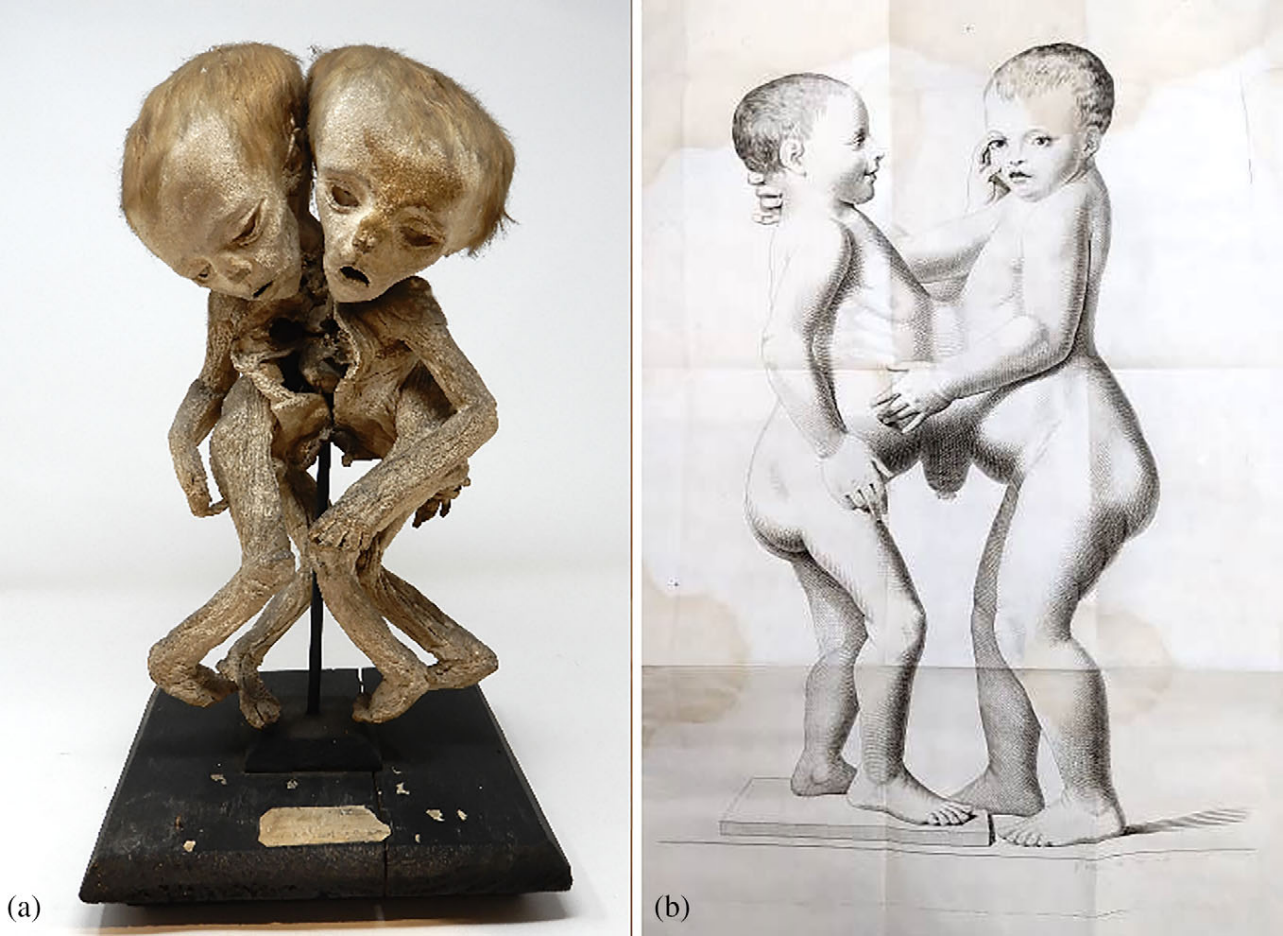
(a) Female conjoined twin specimen corresponding to Fanzago's description from Morgagni Museum. (b) Drawing of the conjoined twins made by Floriano Caldani for Fanzago (1803)

As reported by Fanzago, the family intended to take advantage from the peculiar condition of their children and started to travel through the Veneto region to raise money by exhibiting the conjoined twins to the public. The children were in good health and able to be breastfed until they reached Padua. Shortly afterwards, the conjoined girls presented with seizures and fever possibly as a consequence of continuous exposure to sudden changes in temperature and suboptimal living conditions, first alternately in one and then in the other and finally simultaneous in both. The girls died in Padua on May 31, 1803 (Fanzago, 1803). A specimen corresponding to the description reported by Fanzago is currently present in the collection of the Morgagni Museum (Figure 4).

Pietro Sografi (1756–1815), Floriano Caldani (1772–1836), and Domenico Menato (n.d.) performed a partial autopsy, in order to preserve the body for the Cabinet of Pathological Anatomy. The autopsy showed a single diaphragm, although doubled in size, only two lungs, one in the right body and the other in the left body, two inferiorly joined mediastinal cavities, and two hearts. The liver was located transversely, formed from two lobes almost divided by the falciform ligament with the greater lobe belonging to the smaller girl and the lesser lobe to the larger one. The stomach of the larger girl was located in the region of the left hypochondrium between this part of the liver and the spleen itself. This stomach was found to be diseased and showed an almost eroded aspect, as did the duodenum. The stomach of the smaller girl was located in her left hypochondrium. On closer examination, the posterior part of the liver also showed a partial second liver with its own gallbladder.

A rectum, bladder, uterus and two ovaries were found in the pelvises of both girls. In the largest of the children, however, the rectum was replaced by a blind‐ending intestine with a displaced vermiform appendix. From the bladder of the larger girl, an urachus and the two umbilical arteries arose that merged in the direction of the umbilicus. Each of the two girls had their own pancreases and two kidneys with ureters. Their respective intestines were confusedly arranged but in separate abdominal cavities. Instead, the thymus, which was unique, was placed under the angle created by the sterni of both girls.

Lastly, the two pericardia were joined together but not communicating. In each of them, there was a heart of ordinary size for the age of the twins from which the corresponding arteries and veins arose. The two aortas continued their natural path along the vertebral column of each individual, and from each aorta arose their respective emulgent arteries, while the two caval veins ascended from the upper side of the liver, heading toward the two corresponding hearts.

Fanzago considered these twins to be “*less monstrous*” than others, in having only a few alterations. The twins lived “*for a long time*,” even considering the brief life span of these individuals, mostly due to physical complications related to the malformation.

He stated that if they were born of parents from better socio‐economic backgrounds, which would have kept them in better condition, they would have been assured a longer life.

The condition presented here was diagnosed as omphalopagus (or xiphopagus) conjoined twins, complicated mainly by pulmonary hypoplasia in both and anal atresia in one of the children. Omphalopagus differs from thoracoileopagus in that both twins are joined at their diaphragms and livers but have their own separate hearts and intestinal organs. Since this type of conjoined twinning does not involve vital organs, the chances of initial survival, with or without separation, are fairly favorable, as Fanzago correctly pointed out.

### 3.5 Case 5. Penada and the “monstrous conformation of a lamb” (1803)

**FIGURE 5:**
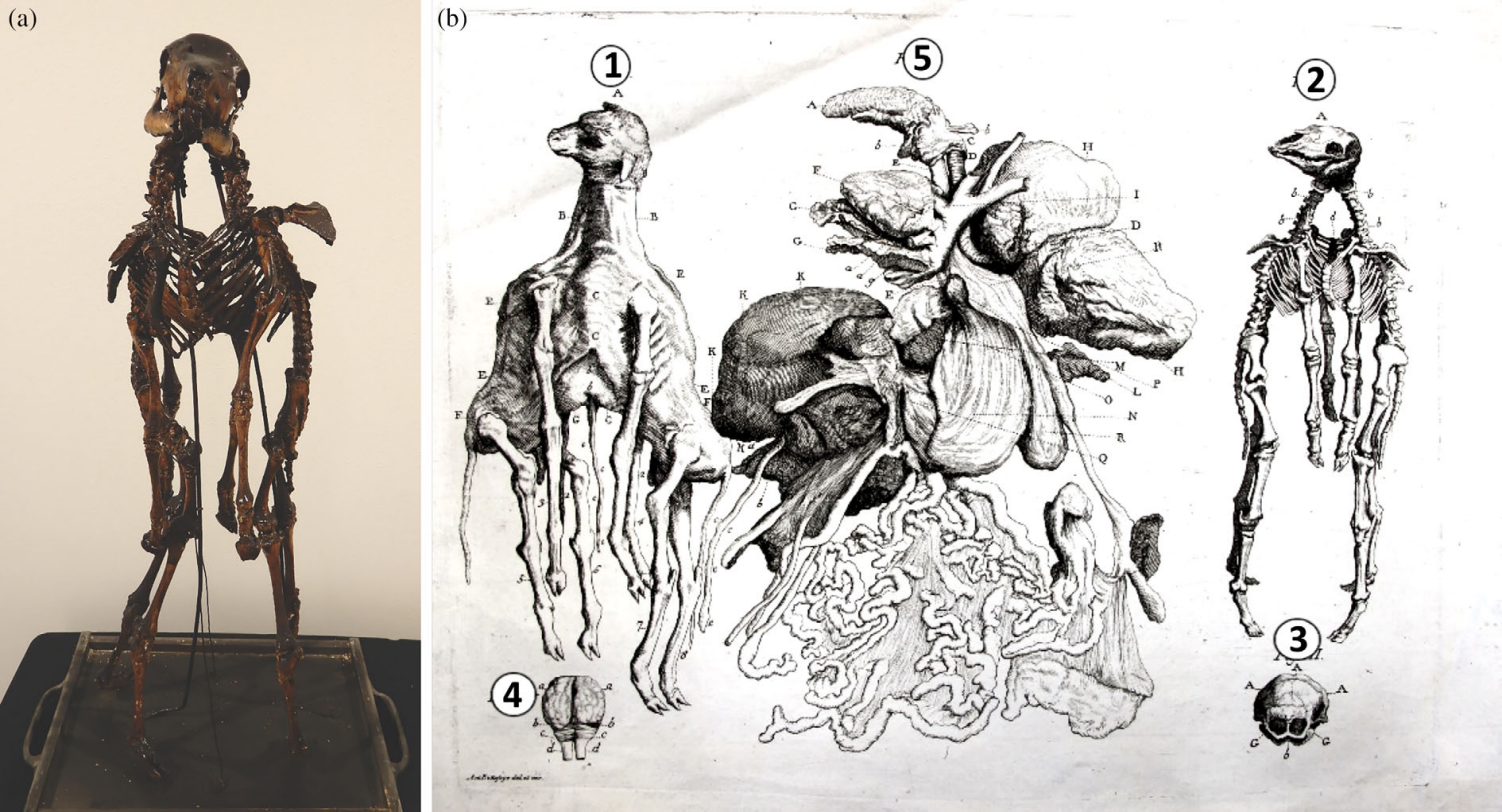
(a) Skeleton of conjoined lamb with single head and two bodies. (b) Drawing made by Penada (1804) and edited by the authors: (1) skinned lamb; (2) skeleton of the lamb; (3) occipital bone with double foramen magnum; (4) brain with double spinal cord; (5) internal organs, from tongue to viscera

Another intriguing case of the Morgagni Museum is the skeleton of a conjoined male lambs studied and preserved by Jacopo Penada and considered as a “*monster with a bizarre conformation*” (Penada, 1804). The specimen is still preserved in the current exhibition of the Museum (Figure 5a).

The lamb was born in Noale near Venice in late January 1803. Given its “monstrous conformation,” the farmers killed it at birth and the body was brought to Penada soon after for the autopsy. He consulted “Lessons of Human Monsters” by Malacarne (1798) to define the class and species of this malformation, and concluded that it belonged to the 10th Class of Polysomy, species “Monocephalus Bisomus,” that is, a single head with two bodies. The lamb was then examined in detail by means of autopsy. The skeleton was prepared and preserved in the old Pathological Museum.

After removal of the skin (Figure 5b‐1), the lamb appeared to have a single neck but with a doubled vertebral column, which were connected to either side of the single head and running down into the two bodies. The two thoraxes were joined ventrally, with two fully formed sterni in an anterior and posterior position respectively and the vertebral columns located on both sides. The upper abdomen was common down to a single umbilicus. There were two distinct pelvises, which hosted the bowels of the two respective lambs. The upper and lower limbs were doubled and connected to their corresponding bodies.

Skeletally (Figure 5b‐2), it was possible to notice that the occipital bone was very voluminous, with two distinct and separate occipital holes (Figure 5b‐3), from which two different spinal cords originated. The skull contained a single brain with distinct major lobes, but the pons, was divided into two equal segments connected with each of the spinal cords (Figure 5b‐4).

Internal organs were also studied during the autopsy (Figure 5b‐5). The animal had a single tongue (Figure 5b‐A), a single hyoid bone (Figure 5b‐B), a single pharynx (Figure 5b‐D), and a single esophagus (Figure 5b‐E) albeit the necks were doubled. All of these parts were naturally located in the ventral aspect of the head. There were four lungs, a couple on each side (right Figure 5b‐G and left Figure 5b‐H), although the right lobes were 10 times smaller than normal, almost rudimentary, but with perfectly formed bronchi, arteries, and veins. There was a single unique heart for both bodies (Figure 5b‐F), normal in size and morphology, located in between the two couples of lungs. The pulmonary artery (Figure 5b‐G) was divided into two main trunks, right and left that were divided again into four other branches for the four lungs. The aorta, instead, originated from the left ventricle, with an extension greater than normal and then divided in each separated neck region. The thoracic aorta had two large trunks, one on the right and one on the left.

The diaphragm was formed by two halves, placed on each side and joined at the center with a proportionately sized tendon on which only one pericardium was attached, enclosing the single heart. The appendages of this diaphragm were doubled and attached to the backs of the two distinct thoraxes in four appendages, two on each side.

Concerning the venous vessels, the pulmonary veins were duplicated, and the inferior caval vein drained all systemic veins. The portal vein was single, as were the liver (Figure 5b‐K), and the proportioned but empty gallbladder (Figure 5b‐A). A single umbilical cord implanted itself in the common placenta.

The spleens, on the other hand, were doubled, one on the right (Figure 5b‐M), resting on one of the first ruminant's stomach (the rumen), and one on the left (Figure 5b‐L), close to the rudiment of another first stomach. The single esophagus, in fact, passed through a hole to the left of the diaphragm, then dilated in the first stomach (Figure 5b‐N), and then continued to the second (reticulum, Figure 5b‐O), the third (the omasum, Figure 5b‐P), and fourth stomach (the abomasum Figure 5b‐Q). These stomachs had a normal morphology on the right side, while on the left there was only a rudiment of the rumen that ended in a blind bag, without the other stomachs, and connected to a rudimentary smaller intestine (Figure 5b‐R). The remainder of the bowels was normally formed in both bodies.

From the detailed description and figures, as well as from studying the skeletonized specimen, we concluded that this case concerns cephalothoracoileopagus conjoined twins, with profound lateral deviation. Consequently, the “posteriorly” located (compound) structures and organs, such as two of the lungs and one of the first stomachs, are subjected to interaction aplasia, causing them to become so underdeveloped that may be hardly recognizable, if at all (Boer et al., 2019). This seems to concern particularly the head area, where the posterior compound face was reduced to what impressed as a broadened occiput. This also explains why only one brain, hyoid, heart, and liver were found during autopsy.

## 4 DISCUSSION

The cases presented here confirm the increasing interest in the development of teratological studies at the University of Padua during the 18th and 19th century. During our research, we rediscover the works of relatively unknown scholars, such as the Bongiovannis, Fanzago, and Penada, who offered interesting etiopathogenic interpretations and thoughts of the malformations that they studied.

Zenone Bongiovanni, reflecting on his case, identified two possible causes for conjoined twinning. One was a fortuitous union of the two embryos in a single pregnancy, due to a pathology that may have corroded the “separation cloth” of the two embryos. The other one concerned a contraction or restriction of the parts that enclosed the fetuses, thereby preventing a proper development. This idea was prompted by the mother's claim of having suffered severe pain in the lower abdomen during gestation. Bongiovanni also suggested a relationship with malnutrition and the poor conditions in which women, who generate such monsters, often lived, implying that this could have been an additional cause for the occurrence of these malformations. Nevertheless, he believed that the occurrence of malformations was a “mystery of Nature” for which man should not compulsorily try to give a specific solution, at the risk of sinning and going against the “divine plan” (Bongiovanni, 1789).

Fanzago believed that his case represented two rudimentary embryos that accidentally became joined in their ventral parts, and gradually grew to give rise to such conditions as a “*Bicorporeal Monster*,” as it may happen in eggs, with two fertilized yolks, as well as in other animals. Like Bongiovanni, he was not keen on drawing further conclusions because he considered malformations as “mysteries of Nature” that should not be disclosed by men (Fanzago, 1803).

Penada, reflecting on generation and on the defects of monstrosities, supposed that most malformations are related to a predisposition or “strange shape” of the ovum or embryo, without providing an explanation of the causes that may lead to this condition. Following his mentor, Malacarne, he refused to believe that imaginations, compressions, and similar concepts caused such malformations but instead supported an organic predisposition and an arrest of organic evolution in the different stages of development. Yet, he still believed the occurrence of malformations to be related to a “divine desire,” which he did not understand, or did he think he would ever do (Penada, 1804).

## 5 CONCLUSION

The medico‐historical review of the literature permitted us to identify and date some of the oldest teratological specimens of the Morgagni Museum of Human Anatomy of Padua. This allowed us to improve our knowledge on the creation of the collection, which is scarce and incomplete for many objects.

These cases and the reports of the scholars who studied them showed that between the end of the 18th and the beginning of the 19th century there was a gradual progression in the attempts to understand congenital malformations. Preservation of these specimens was relevant to continue studying their conditions to increasing the knowledge about their etiopathogenesis of congenital anomalies, and to slowly detach this knowledge from ancient ideas of mere supernatural origins of these birth defects.

Additionally, the case presented by Fanzago (1803) also gives insight in the perception of lay men being confronted with congenital malformations and the impact on their daily life. In particular, this concerns the birth of conjoined individuals and their chances of survival, even in cases potentially compatible with life, which were often disadvantaged by poverty and archaic misconceptions toward these birth defects.

The present day rationale in the etiopathogenetic formation of conjoined twins remains matter for ongoing debate. Besides the fission and fusion theory, a third concept, initially propagated by Spencer (2003), has been discussed recently and can be interpreted as “crowding.” This “crowding” theory propagates a novel concept of how conjoined entities might arise and how their ultimate phenotypes can be generated and explained (Boer et al., 2019, 2021).

The crowding theory states that the origin of conjoined entities lies in the formation of duplicated (or triplicated) embryological primordia within a single overarching embryological disk, thereby showing overlap with the fission theory in which embryological structures partially divide and subsequently influence each other outgrowth. Furthermore, both the fission and crowding theory state that the ultimate conjunction patterns reside in the initial location of primordial duplications. Besides the formation of duplications, mechanical influences (e.g., interaction aplasia and neo‐axial orientation) are responsible for the ultimate phenotype of these peculiar twins. Interestingly, these novel concepts unconsciously started in the past and built up on the “older” reflections that are described in this article, showing that past epochs and their etiopathogenetic thoughts are the basis for our current understanding in unraveling the genesis of congenital anomalies. Therefore, these ancient specimens and their historical treatises should not be overlooked.

## CONFLICT OF INTEREST

The authors declare that there is no conflict of interest.

## Data Availability

Data sharing not applicable to this article as no datasets were generated or analysed during the current study.
